# The Abundance and Function of Neutrophils in the Endometriosis Systemic and Pelvic Microenvironment

**DOI:** 10.1155/2023/1481489

**Published:** 2023-01-31

**Authors:** Xinlei Wang, Yan Jia, Danyang Li, Xiaopin Guo, Zhengjie Zhou, Mingran Qi, Guoqiang Wang, Fang Wang

**Affiliations:** ^1^Department of Pathogeny Biology, College of Basic Medical Sciences, Jilin University, Changchun 130021, China; ^2^Department of Gynecology and Obstetrics, The Second Hospital of Jilin University, Changchun 130041, China

## Abstract

Endometriosis is a common inflammatory illness in which endometrial tissue grows outside the uterine cavity. Immune dysfunction is now widely acknowledged as the primary cause of endometriosis. The immune cell population represented by neutrophils is thought to play an essential role in the etiology, pathophysiology, and associated clinical outcome. There is growing evidence that neutrophils have a role in chronic and aseptic inflammatory diseases, and endometriosis patients have increased levels of neutrophils in plasma, peritoneal fluid, and ectopic endometrium. Here, we sought to review the function of neutrophils in the pathogenesis of endometriosis, with an emphasis on the role of neutrophils in regulating endometrial angiogenesis and the local inflammatory microenvironment.

## 1. Introduction

Endometriosis is a chronic inflammatory disease defined by the presence of endometrium-like tissue lesions outside of the uterus that the prevalence varies from 6% to 10% [1–3]. The common symptoms of endometriosis are pelvic pain and/or infertility, when also includes some asymptomatic cases [4]. Endometriosis has a multifactorial etiology; the most widely recognized theory is retrograde menstruation, which occurs when live endometrial cells reflux past the fallopian tubes into the pelvis during menstruation [5]. Although retrograde menstruation is a fairly common phenomenon, endometriosis affects only a tiny number of women. There is substantial evidence that the immune system plays a role in the pathophysiology and clinical symptomatology of endometriosis, whereas genetics and environment are the major causes. Immunological dysfunction is thought to hinder menstrual debris from being removed, causing it to stick to pelvic tissues and develop a new blood supply, resulting in endometriotic lesions, which cause local inflammatory responses, scarring, and pain [6, 7]. The anatomical distortion caused by scarring leads to infertility, and local inflammation affects oocyte quality and early embryonic development, as well as the unsuitable endometrial environment for embryo implantation [8]. Multiomics-based analysis suggests the presence of altered steroid hormone signaling, antigen presentation, upregulation of lymphocyte activation, and cytokine-induced and inflammatory pathways in endometriotic lesions [9–11]. There is an altered proinflammatory immune milieu in the microenvironment of endometriosis lesions. Although some studies have provided an overview of the impact of innate and adaptive immune abnormalities on endometriosis pathophysiology, there is still a knowledge gap regarding the altered functional properties of specific immune cells and how this may lead to changes in endometriosis phenotype and disease symptoms.

Neutrophils (NT) have traditionally been thought to constitute the primary immune response to infection and tissue injury, defending against invading pathogens by phagocytosis, degranulation, and neutrophil extracellular traps (NETs) [12, 13]. However, there is mounting evidence that neutrophils are a homogeneous population of cells with a variety of particular functions, and that various subpopulations of these cells have immunomodulatory roles and are linked to cancer and other inflammatory illnesses [13, 14]. Importantly, the presence of tissue-based populations of neutrophils that are both colonized and newly infiltrated and acquire specific functions or phenotypes depending on the tissue microenvironment is observed [13, 15, 16]. The local microenvironment can influence neutrophil polarization, and the development of sterile and chronic inflammatory diseases, including endometriosis [1], inflammatory bowel disease (IBD) [17], metabolic syndrome [18], and atherosclerosis [19], is associated with neutrophil recruitment and activation [20, 21]. In contrast, neutrophil populations in the tumor microenvironment are essentially determined by their functional phenotype, with neutrophils performing proinflammatory/anti-inflammatory and protumor/antitumor roles [20, 22]. Neutrophils and their released cytokines have been demonstrated to be higher in the circulatory system and peritoneal fluid of endometriosis patients, promoting endometriotic cell proliferation, invasion, and angiogenesis [11, 23–25]. In this review, we focus on the involvement of neutrophils in endometriosis pathophysiology, including the abundance, function, and activation status of the neutrophil population, as well as the role of neutrophil heterogeneity and plasticity in endometriosis lesion formation and angiogenesis.

## 2. Methods

On PubMed and Medline, a thorough literature search was conducted for articles published up to 30 November 2022. Endometriosis, neutrophils, neutrophil extracellular traps, pelvic microenvironment, angiogenesis, inflammation, and cytokines were all combined as search terms. The literature search did not use linguistic filtering or publication deadline restrictions. The evaluation of the results included both human and animal studies. Editorials, communications, and notes are not included. To find pertinent studies, reference lists of retrieved studies were manually examined, and 322 articles were found after a review of the literature in the PubMed database. After extensive research, a total of 19 articles were collected.

## 3. Endometrial Neutrophils in Menstruation

Menstruation is an inflammatory process accompanied by tissue breakdown and bleeding that occurs at the end of each normal menstrual cycle [26]. The loss of progesterone and estrogen in the nonconceptive cycle causes a decrease in prostaglandin metabolism and a loss of defense against reactive oxygen species (ROS). Increased ROS causes the release of NF*κ*B, which causes the induction of inflammatory signals and the release of proinflammatory mediators such as prostaglandins, cytokines, chemokines, and matrix metalloproteinase (MMP) synthesis, which causes neutrophil recruitment and phenotypic changes. This, combined with the hypoxic environment induced by prostaglandin action, leads to the rapid destruction of the extracellular matrix, which in turn causes menstrual tissue rupture and hemorrhage [26–29].

**Figure 1:**
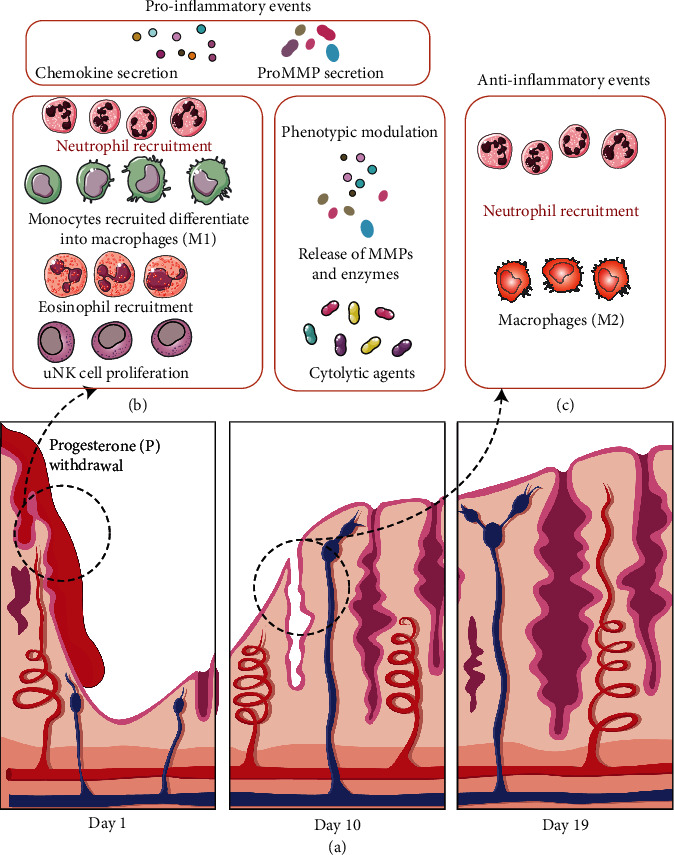
Inflammatory events in menstruation. (a) Ovarian-derived estradiol and progesterone play a significant role in the structure and shape of the endometrium throughout the menstrual cycle. (b) Progesterone withdrawal leads to the release of chemokines into the surrounding tissues and the recruitment of neutrophils into the endometrium. Macrophages may have a proinflammatory (M1) phenotype in the early stages, and their phagocytic clearance of apoptotic cells is required to reduce local inflammation. Eosinophils are also recruited, while local mast cells become highly active and uterine natural killer (uNK) cells proliferate and release cytolytic chemicals. As these cells undergo a phenotypic shift, MMPs and degradative enzymes are released, stimulating the MMP activation cascade and driving ECM breakdown and endometrial rupture. (c) After menstruation, macrophages remove cellular debris while neutrophils of different phenotypes assist in the remodeling and repair of the functional endometrial layer.

Neutrophils are barely detectable in the normal endometrium throughout most of the menstrual cycle, but their numbers skyrocket right before the onset of menstruation, accounting for 6–15% of the total number of cells in the tissue when neutrophils are most abundant. Chemokines released by mast cells (MC), macrophages (Mø), and other cells may cause an increase in endometrial neutrophils during menstruation [26]. However, quantifying the endometrial neutrophil population is very challenging due to the influence of the ovarian endocrine cycle and the great heterogeneity of immune cell distribution patterns in the endometrium. Neutrophils contribute to endometrial function, not only by killing but also by restoring endometrial tissue. Progesterone withdrawal corresponds with neutrophil recruitment throughout the premenstrual period [30]. Neutrophils contain high levels of matrix metalloproteinase (MMP) and may activate MMP in situ [31], which leads to endometrial rupture during menstruation. Neutrophils recruited during menstruation are an integral part of the endometrial repair process. The absence of neutrophils impedes endometrial repair in a mouse model that resembles menstruation, demonstrating the importance of the local endometrial immune cell population [32]. Endometrial neutrophils secrete IFN-*γ*, vascular endothelial growth factor (VEGF), and IL-17, which are involved in cell proliferation, angiogenesis, and immune response, all of which are necessary for implantation and/or pregnancy maintenance [33, 34]. The inflammatory events throughout the normal menstrual cycle are summarized in Figure 1.

## 4. Neutrophils in Endometriosis Eutopic Endometrium

**Figure 2:**
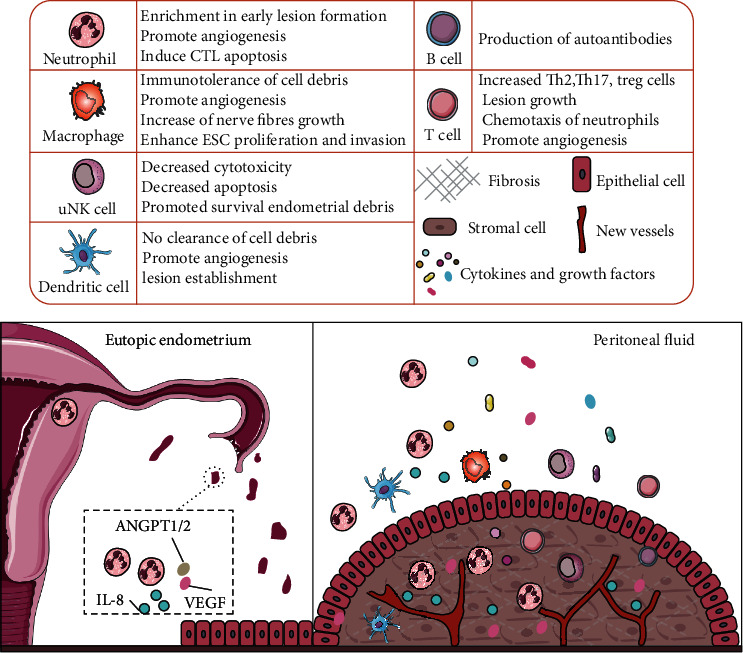
The function of immune cells in the microenvironment of endometriotic lesions. During menstruation, according the theory of retrograde menstruation, live endometrial debris first returns to the peritoneal cavity, containing a large number of neutrophils, cytokines (IL-8), and angiogenic factors (VEGF and ANGPT1/2), which interact with various infiltrative immune cell populations and promote endometrial cell adhesion and cell proliferation. Under the influence of systemic and local abdominal immune dysfunction, immune cells and immune mediators promote the establishment and survival of lesions. The table above outlines the role of each immune cell type in the pathophysiology of endometriosis.

Various immune cells create a proangiogenic immune microenvironment in endometriosis, endometrial infiltrating macrophages, and neutrophils is an important source of cytokines and chemokines. Various immune cell populations invading the endometriotic lesion interact with epithelial, mesenchymal, and endothelial cells, contributing to the creation and evolution of endometriotic lesions inside the peritoneal cavity, as shown in Figure 2. Notably, several mechanisms by which neutrophils contribute to endometriosis have been proposed, but most of them are based on the expression of neutrophil-associated cytokines and chemokines. VEGF is a significant angiogenic agent in the endometrium throughout the menstrual cycle, ovulation, and tissue regeneration, with macrophages and neutrophils being the main sources of VEGF expression in the endometrium [35]. Interleukin-8 (IL-8) is a potent angiogenic cytokine that causes neutrophil chemotaxis. IL-8 levels are higher in the normal endometrium during the secretory phase compared to the proliferative phase. In women with endometriosis, IL-8 levels are higher in the early proliferative and late secretory phases of the eutopic endometrium, which increases neutrophil recruitment [36, 37]. Increased expression of VEGF and angiopoietin 1 and 2 (ANGPT1/2) in the eutopic endometrium causes dysregulated angiogenic activity [38], and structural and/or functional differences in the eutopic endometrium may play a role in endometrial cells entering the peritoneal cavity to form endometriosis lesions [39, 40]. Thus, increased neutrophils and VEGF, which are involved in the etiology of endometriosis, induce a vicious cycle of endometrial cell adhesion, cell proliferation, and further IL-8 release, necessitating additional research into the role of neutrophils in endometrial circulation.

Endometriosis lesions are characterized by dense vascularization, and the efficacy of antiangiogenic therapy has been tested in a small number of animal studies [41]. Resveratrol, a polyphenolic compound with antitumor, anti-inflammatory, antioxidant, and anti-angiogenic properties [42], was recently tested in a human clinical trial to reduce VEGF and TNF-*α* expression in postoperative eutopic endometrium of patients with endometriosis (stage III-IV), and thus, antiangiogenic therapy may be a successful approach for the treatment of endometriosis [43].

## 5. Neutrophils and Related Cytokines in the Peritoneal Microenvironment

The inflammatory peritoneal environment contributes to the colonization of endometriosis lesions, and the peritoneal fluid contains high levels of cytokines and growth factors [44], such as interferon-gamma-induced protein-10 (CXCL10), IL-8, and chemokine growth-regulated-alpha (GRO-*α*) [45–47]. Neutrophils in the ectopic endometrium are a plentiful source of VEGF, which directly contributes to the high VEGF levels in the peritoneal fluid [48]. The presence of neutrophils and VEGF can establish a microenvironment that promotes inflammation and angiogenesis, and surgical excision of endometriotic lesions reduces serum VEGF levels, indicating that peritoneal lesions have a significant influence on the microenvironment [49]. It has been reported that the frequency of neutrophils in the peritoneal fluid is higher in women with endometriosis in stages I and II but not in stage III and IV [23]. Additionally, it has been demonstrated that all phases of endometriosis had higher peritoneal fluid neutrophil percentages than controls [24]. The possible reasons for this difference are due to differences in the techniques used to collect peritoneal fluid and differences in the methods of neutrophil detection.

Interleukin 8 (IL-8), a cytokine that induces neutrophil chemotaxis and promotes angiogenesis and cell proliferation, is elevated in the peritoneal fluid of patients with endometriosis, and its level correlates with the severity of the disease [11, 24, 36]. Increased IL-8 levels in the endometriosis peritoneal microenvironment can upregulate Fas-ligand (FasL) expression in endometrial cells and induce cytotoxic T lymphocyte apoptosis, potentially establishing a local immune tolerance environment for ectopic endometrial implantation [50]. neutrophil-activating peptide 78 (ENA-78), a neutrophil chemokine, is significantly elevated in peritoneal fluid from endometriosis patients, not only by stimulating neutrophils to secrete growth factors and cytokines but also by directly promoting endometrial stromal cell proliferation, which may play a role in the growth and maintenance of ectopic endometrial tissue [51–54]. Furthermore, in cultured endometrial stromal cells in vitro, the addition of IL-1*β* or TNF-*α* with IFN-*γ* promoted the release of large amounts of ENA-78 and IL-8 [54, 55]. Thus, the endometriotic lesions are established in a highly complex and dynamic peritoneal microenvironment of inflammation, angiogenesis mediated by neutrophils and other immune cells.

## 6. Neutrophil Extracellular Traps (NETs) in Endometriosis

The ability of NETs to host defenses was initially studied, but more recently, their pathogenic potential has been studied, including their involvement in controlling sterile inflammation, vascular obstruction, and tissue damage [56]. And endometriosis is associated with higher levels of NETs in peritoneal fluid compared to controls [57]. Circulating plasma NET levels were significantly higher in deeply infiltrated endometriosis, indicating a possible role for NETs in endometriosis pathogenesis and confirming it as a chronic sterile inflammatory disease [58]. However, peritoneal fluid from endometriosis patients did not stimulate the release of NETs from healthy neutrophils, and more research is needed to investigate the connection between NETs and endometriosis pathogenesis [57].

## 7. Neutrophils and Related Cytokines in Ectopic Endometriosis Lesions

Compared to normal endometrium, ectopic endometrial lesions are heterogeneous and ectopic endometrium has a microenvironment that encourages neutrophil recruitment. In ectopic endometrium, the percentage of neutrophils (myeloperoxidase positive, MPO+) was found to be considerably higher (*P* = 0.0209) [11]. As neoangiogenesis is a requirement for intraperitoneal endometriosis lesions to survive, numerous angiogenic involving VEGF and other factors and proinflammatory cytokines, including IL-8 and CXCL10, are overexpressed in ectopic endometriosis lesions [34, 48]. Ovarian endometriosis is the most common site of ectopic endometrium [1]. When compared to control follicular cyst fluid, ovarian endometriosis cyst fluid contains higher levels of VEGF and IL-8, which may stimulate neutrophil recruitment and angiogenesis, allowing ectopic endometrial cell implantation into the ovary [59].

Moreover, interleukin 17A (IL-17A), a proangiogenic factor produced primarily by Th17 cells but also recently discovered to be expressed by neutrophils [60, 61], is found to be higher in ectopic endometriosis than in eutopic endometriosis and correlates with the American Society for Reproductive Medicine stage of endometriosis [62]. And IL-17A produced by neutrophils stimulates endometrioma stromal cells to secrete growth-regulated oncogene-*α* (GRO-*α*), a member of the C-X-C family of chemokines, thereby recruiting more neutrophils and inducing the formation and maintenance of endometriosis [63, 64]. When compared to matched eutopic or normal endometrium, endometriosis lesions exhibit upregulation of numerous genes for cytokines, chemokines, adhesion molecules, and damage-associated molecular patterns (DAMPs) that impact neutrophil recruitment, function, and survival [11, 65, 66] (Table 1).

## 8. Neutrophils in Plasma of Endometriosis

The chronic proinflammatory environment in the peritoneum promotes endometriosis lesion colonization and disease progression, which is associated with elevated neutrophils both circularly and locally. The ratio of neutrophils/lymphocytes in peripheral blood of patients with endometriosis was increased [67] and also found that adding plasma and peritoneal fluid from patients with endometriosis to neutrophils cultured in vitro significantly reduced the percentage of neutrophils apoptotic cells, resulting in an increased number of neutrophils in endometriosis patients' blood [23]. The transcriptome of neutrophils extracted from the peripheral blood of endometriosis patients and healthy controls indicated that the innate immune response, cytokine-mediated signaling pathway, and inflammatory responses were the most significantly changed biological processes [11]. One of the main functions of neutrophils is phagocytosis [13], and in patients with endometriosis, neutrophil phagocytosis in the peripheral blood was diminished; however, neutrophil phagocytosis in these patients improved dramatically after 7 days postoperatively [68]. Compared to healthy controls, IL-17A concentrations were significantly increased in patients with endometriosis and decreased in plasma after excision of the lesion, reinforcing the association of IL-17A with the pathophysiological process of endometriosis [63]. YKL-40 is an inflammatory biomarker that is secreted by activated macrophages and neutrophils [69]. Serum YKL-40 levels are higher in women with endometriosis and are positively correlated with disease stage, suggesting that YKL-40 may serve as a marker for endometriosis [70]. In conclusion, circulating blood neutrophils and cytokines are elevated in patients with endometriosis and may be involved in the pathophysiology.

**Figure 3:**
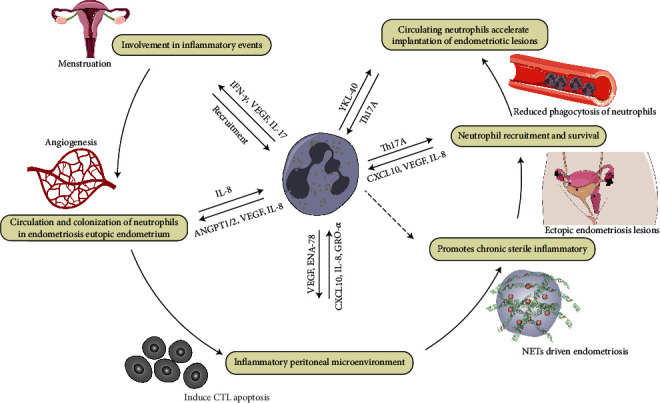
Mechanisms of neutrophil-mediated pathophysiology of endometriosis. The number of neutrophils increases significantly in the premenstrual period and participates in the shedding and repair of the endometrium. Endometriosis eutopic endometrium has a high neutrophil count, which overexpresses VEGF, IL-8, and ANGPT1/2, resulting in dysregulation of angiogenic activity and further tissue shedding to form ectopic endometrial lesions, shaping the inflammatory peritoneal microenvironment. A vicious cycle of recruiting neutrophils, secreting cytokines and growth factors, and promoting apoptosis of cytotoxic T cells is further formed. In this process, the increase of neutrophils in the circulating blood is accompanied by the weakening of phagocytosis, and pathophysiological pathways like NETs are also involved, generating a highly complex and dynamic microenvironment. IFN-*γ*: interferon-*γ*; VEGF: vascular endothelial growth factor; IL-17: interleukin-17; IL-8: interleukin-8; ANGPT1/2: angiopoietin 1 and 2; ENA-78: epithelial neutrophil activating peptide-78; CXCL10: interferon-gamma-induced protein-10; GRO-*α*: chemokine growth-regulated-alpha.

An enhanced proinflammatory state of circulating neutrophils was identified in patients with endometriosis. Low-density granulocytes (LDGs), a subset of circulating proinflammatory neutrophils, were first described in systemic lupus erythematosus [71, 72]. Recent research has revealed that LDGs are related to a variety of autoimmune and inflammatory diseases. LDGs also have the ability to synthesize a large number of proinflammatory cytokines, including IFN-*γ* and TNF-*α* [71, 73, 74]. It can be speculated that the abundance of LDGs in the circulation of endometriosis may increase. However, more research is needed to confirm the abundance and pathophysiological role of LDGs in the peripheral blood and pelvic microenvironment of endometriosis patients. The pathophysiological mechanism of neutrophils involved in endometriosis is shown in Figure 3.

## 9. Neutrophils in Mouse Models of Endometriosis

Because there are no animal models of spontaneous menstruation (except for Old World primates) that can recreate human endometriosis, as well as the disease's heterogeneity and the effect of the immune-endocrine axis, creating endometriosis models in animals is challenging, and most animal studies are based on mice models. In a syngeneic mouse model of endometriosis established through uterine horn peritoneal transplantation, neutrophils were significantly enriched at 24–72 hours of lesion establishment, with a peak at day 2–3 days, indicating that neutrophils may play a role in the early response of endometrial tissue to the local peritoneal environment [11, 75]. In support of this, depletion of neutrophils with neutralizing antibodies to granulocyte receptor-1 (Gr-1) [76] or lymphocyte antigen 6 complex, locus G (Ly6G) [77] reduced the formation of endometriotic lesions in mice [11, 76, 78]. In the early stages of endometriotic lesion formation, neutrophils enhance lesion vascularization by increasing the production of local and systemic cytokines and angiogenic mediators [11, 75, 79]. In peritoneal fluid and ectopic endometrium of endometriosis model mice, neutrophil counts and levels of granulocyte colony-stimulating factor (G-CSF) and interleukin-6 (IL-6) were significantly increased. G-CSF and IL-6 regulate neutrophils through STAT3 pathways and alter the expression of angiogenesis-related genes (Mmp9, Bv8, and Trail), which are involved in the establishment of early endometriosis [78]. Neovascularization is essential for the formation of endometriosis lesions, and inhibition of endometriosis angiogenesis with COX-2 inhibitors was shown to reduce neutrophil infiltration in endometriosis lesions [41].

Endometriosis is known to be estrogen-dependent, and local estradiol biosynthesis in endometriotic lesions combines with peritoneal inflammation in the peritoneal cavity to form an abnormal immune-endocrine microenvironment [1]. In breast tissue, estrogen function supports neutrophil infiltration and neutrophil-mediated adipocyte regeneration [80]. In the early phases of endometriosis, estrogen (E2) and estrogen receptor (ER) are essential for immunological regulation and angiogenesis [79], and the physiological connection between estrogen-induced modifications in neutrophil function and endometriosis pathogenesis is of particular interest.

## 10. Neutrophils in Endometriosis Clinical Application

Neutrophils are mostly used in endometriosis to diagnose or differentiate the severity of the disease depending on the number of neutrophils in the peripheral blood. The neutrophil-to-lymphocyte ratio (NLR) has been proposed as a diagnostic marker for endometriosis, and when combined with serum CA-125, it can improve diagnostic sensitivity and screen for more minor-to-mild diseases [44, 81]. NLR is a valuable adjunct in the diagnosis of advanced endometriosis, as well as in the diagnosis for patients with negative serum CA-125 [82]. However, some studies did not find a correlation between NLR and endometriosis [83, 84]. Further studies are still needed to explain these differences. In terms of therapeutic strategies, neutrophils alone or in combination with CA125 are potential targets for the diagnosis of endometriosis.

## 11. Conclusions and Prospects

Retrograde menstruation occurs in most women, but only a fraction of women develops endometriosis. It has been hypothesized that women with congenital and adaptive immune disorders are more likely to develop endometriosis. Ultimately, the influence of estrogen, immune dysfunction, and angiogenesis is considered to be important in the development of endometriosis. Ectopic endometrial lesions lead to rapid neutrophil recruitment, and neutrophil infiltration contributes to early angiogenesis in the ectopic endometrial inflammatory microenvironment, which forms a positive feedback. Here, we review the behavior of neutrophils in the different settings and locations of endometriosis, where neutrophils and associated immune mediators promote lesion establishment and survival, contributing to the understanding of the potential role of neutrophils in disease pathogenesis and pathophysiology.

However, based on present evidence, we are unable to conclude whether neutrophil phenotype and function contribute a role in the formation and progression of endometrial lesions. The bulk of neutrophil-based research to date has been observational, with neutrophils or their cytokine products assessed in the systemic circulation and peritoneal fluid alone or in combination. The involvement of neutrophils in endometriosis is expected to be better understood using high-throughput sequencing platforms, single-cell genomics, and multiomics techniques.

## Figures and Tables

**Table 1:** Cytokines and chemokines associated with neutrophil recruitment in endometriosis.

Species	Alterations	Ectopic vs. eutopic endometriosis	Plasma	Peritoneal fluid
Human	Increased	IL-8 [11]. GM-CSF and IL-15 [85]. EG-VEGF [40]. VEGF	IL-8 [86]. YKL-40 [70].	HNP 1–3, IL-6, and IL-8 [24]. MCP-1, IL-6, and IL-8 [86]. VEGF and IL-6 [45]. CXCL10 and IL-8 [46]. GRO-*α* [47].
	Decreased	G-CSF, MIP-1*β*, IP-10, FGF-basic, IL-1ra, IL-5, and IL-7 [85].	IFN-*γ* [87].	—
Mice	Increased	—	G-CSF, CXCL2, CCL2, CCL11, G-CSF, CXCL2, CCL2, and CCL11 [11]. G-CSF and IL-6 [78].	G-CSF, CXCL1, CCL11, CCL3, TNF-*α*, IL-6, IL-4, and VEGF [11]. CCL2, G-CSF, IL6, and VEGF [79]. GM-CSF, IL-10, and IL-17 [79]. G-CSF and IL-6 [78].
	Decreased	—	—	—

IL-8: interleukin-8; GM-CSF: granulocyte-macrophage colony-stimulating factor; G-CSF: granulocyte colony-stimulating factor; MIP-1*β*: macrophage inflammatory protein 1*β*; HNP1-3: human neutrophil peptides 1-3; IP-10: interferon-inducible protein 10; FGF-basic: basic fibroblast growth factor; IL-1ra: IL-1 receptor antagonist; EG-VEGF: endocrine gland-derived VEGF; GRO-*α*: chemokine growth-regulated-alpha.

## References

[1] Zondervan K. T., Becker C. M., Koga K., Missmer S. A., Taylor R. N., Viganò P. (2018). Endometriosis. *Nature Reviews. Disease Primers*.

[2] Agarwal S. K., Chapron C., Giudice L. C. (2019). Clinical diagnosis of endometriosis: a call to action. *American Journal of Obstetrics and Gynecology*.

[3] Wei Y., Liang Y., Lin H., Dai Y., Yao S. (2020). Autonomic nervous system and inflammation interaction in endometriosis-associated pain. *Journal of Neuroinflammation*.

[4] Chapron C., Marcellin L., Borghese B., Santulli P. (2019). Rethinking mechanisms, diagnosis and management of endometriosis. *Nature Reviews. Endocrinology*.

[5] Viganò P., Parazzini F., Somigliana E., Vercellini P. (2004). Endometriosis: epidemiology and aetiological factors. *Best Practice & Research. Clinical Obstetrics & Gynaecology*.

[6] Halme J., Hammond M. G., Hulka J. F., Raj S. G., Talbert L. M. (1984). Retrograde menstruation in healthy women and in patients with endometriosis. *Obstetrics and Gynecology*.

[7] Vallvé-Juanico J., Houshdaran S., Giudice L. C. (2019). The endometrial immune environment of women with endometriosis. *Human Reproduction Update*.

[8] Carson S. A., Kallen A. N. (2021). Diagnosis and management of infertility: a review. *JAMA*.

[9] Houshdaran S., Nezhat C. R., Vo K. C., Zelenko Z., Irwin J. C., Giudice L. C. (2016). Aberrant endometrial DNA methylome and associated gene expression in women with endometriosis. *Biology of Reproduction*.

[10] Sbracia M., Valeri C., Antonini G., Biagiotti G., Pacchiarotti A., Pacchiarotti A. (2016). Fas and Fas-ligand in eutopic and ectopic endometrium of women with endometriosis: the possible immune privilege of ectopic endometrium. *Reproductive Sciences*.

[11] Symons L. K., Miller J. E., Tyryshkin K. (2020). Neutrophil recruitment and function in endometriosis patients and a syngeneic murine model. *The FASEB Journal*.

[12] Ley K., Hoffman H. M., Kubes P. (2018). Neutrophils: new insights and open questions. *Sci Immunol*.

[13] Silvestre-Roig C., Fridlender Z. G., Glogauer M., Scapini P. (2019). Neutrophil diversity in health and disease. *Trends in Immunology*.

[14] Rosales C. (2018). Neutrophil: a cell with many roles in inflammation or several cell types?. *Frontiers in Physiology*.

[15] Liew P. X., Kubes P. (2019). The neutrophil's role during health and disease. *Physiological Reviews*.

[16] Christoffersson G., Phillipson M. (2018). The neutrophil: one cell on many missions or many cells with different agendas?. *Cell and Tissue Research*.

[17] Li T., Wang C., Liu Y. (2020). Neutrophil extracellular traps induce intestinal damage and thrombotic tendency in inflammatory bowel disease. *Journal of Crohn's & Colitis*.

[18] Barden A., Shinde S., Tsai I. J. (2019). Effect of weight loss on neutrophil resolvins in the metabolic syndrome. *Prostaglandins, Leukotrienes, and Essential Fatty Acids*.

[19] Döring Y., Soehnlein O., Weber C. (2017). Neutrophil extracellular traps in atherosclerosis and atherothrombosis. *Circulation Research*.

[20] Giese M. A., Hind L. E., Huttenlocher A. (2019). Neutrophil plasticity in the tumor microenvironment. *Blood*.

[21] Salamonsen L. A., Lathbury L. J. (2000). Endometrial leukocytes and menstruation. *Human Reproduction Update*.

[22] Filippi M. D. (2019). Neutrophil transendothelial migration: updates and new perspectives. *Blood*.

[23] Tariverdian N., Siedentopf F., Rücke M. (2009). Intraperitoneal immune cell status in infertile women with and without endometriosis. *Journal of Reproductive Immunology*.

[24] Milewski Ł., Dziunycz P., Barcz E. (2011). Increased levels of human neutrophil peptides 1, 2, and 3 in peritoneal fluid of patients with endometriosis: association with neutrophils, T cells and IL-8. *Journal of Reproductive Immunology*.

[25] Ottolina J., Bartiromo L., Dolci C. (2020). Assessment of coagulation parameters in women affected by endometriosis: validation study and systematic review of the literature. *Diagnostics (Basel)*.

[26] Evans J., Salamonsen L. A. (2012). Inflammation, leukocytes and menstruation. *Reviews in Endocrine & Metabolic Disorders*.

[27] Critchley H. O. D., Maybin J. A., Armstrong G. M., Williams A. R. W. (2020). Physiology of the endometrium and regulation of menstruation. *Physiological Reviews*.

[28] Gellersen B., Brosens J. J. (2014). Cyclic decidualization of the human endometrium in reproductive health and failure. *Endocrine Reviews*.

[29] Shen H. H., Zhang T., Yang H. L. (2021). Ovarian hormones-autophagy-immunity axis in menstruation and endometriosis. *Theranostics*.

[30] Armstrong G. M., Maybin J. A., Murray A. A. (2017). Endometrial apoptosis and neutrophil infiltration during menstruation exhibits spatial and temporal dynamics that are recapitulated in a mouse model. *Scientific Reports*.

[31] Selvais C., Gaide Chevronnay H. P., Lemoine P. (2009). Metalloproteinase-dependent shedding of low-density lipoprotein receptor-related protein-1 ectodomain decreases endocytic clearance of endometrial matrix metalloproteinase-2 and -9 at menstruation. *Endocrinology*.

[32] Kaitu'u-Lino T. J., Morison N. B., Salamonsen L. A. (2007). Neutrophil depletion retards endometrial repair in a mouse model. *Cell and Tissue Research*.

[33] Yeaman G. R., Collins J. E., Currie J. K., Guyre P. M., Wira C. R., Fanger M. W. (1998). IFN-gamma is produced by polymorphonuclear neutrophils in human uterine endometrium and by cultured peripheral blood polymorphonuclear neutrophils. *Journal of Immunology*.

[34] Fan X., Krieg S., Kuo C. J. (2008). VEGF blockade inhibits angiogenesis and reepithelialization of endometrium. *The FASEB Journal*.

[35] McLaren J. (2000). Vascular endothelial growth factor and endometriotic angiogenesis. *Human Reproduction Update*.

[36] Arici A. (2002). Local cytokines in endometrial tissue: the role of interleukin-8 in the pathogenesis of endometriosis. *Annals of the New York Academy of Sciences*.

[37] Sikora J., Smycz-Kubańska M., Mielczarek-Palacz A., Kondera-Anasz Z. (2017). Abnormal peritoneal regulation of chemokine activation—the role of IL-8 in pathogenesis of endometriosis. *American Journal of Reproductive Immunology*.

[38] Di Carlo C., Bonifacio M., Tommaselli G. A., Bifulco G., Guerra G., Nappi C. (2009). Metalloproteinases, vascular endothelial growth factor, and angiopoietin 1 and 2 in eutopic and ectopic endometrium. *Fertility and Sterility*.

[39] Bourlev V., Volkov N., Pavlovitch S., Lets N., Larsson A., Olovsson M. (2006). The relationship between microvessel density, proliferative activity and expression of vascular endothelial growth factor-A and its receptors in eutopic endometrium and endometriotic lesions. *Reproduction*.

[40] Lee K. F., Lee Y. L., Chan R. W. (2010). Up-regulation of endocrine gland-derived vascular endothelial growth factor but not vascular endothelial growth factor in human ectopic endometriotic tissue. *Fertility and Sterility*.

[41] Rudzitis-Auth J., Nickels R. M., Menger M. D., Laschke M. W. (2018). Inhibition of cyclooxygenase-2 suppresses the recruitment of endothelial progenitor cells in the microvasculature of endometriotic lesions. *The American Journal of Pathology*.

[42] Li H., Xia N., Hasselwander S., Daiber A. (2019). Resveratrol and vascular function. *International Journal of Molecular Sciences*.

[43] Khodarahmian M., Amidi F., Moini A. (2021). A randomized exploratory trial to assess the effects of resveratrol on VEGF and TNF-*α* 2 expression in endometriosis women. *Journal of Reproductive Immunology*.

[44] Cho S., Cho H., Nam A. (2008). Neutrophil-to-lymphocyte ratio as an adjunct to CA-125 for the diagnosis of endometriosis. *Fertility and Sterility*.

[45] Mahnke J. L., Dawood M. Y., Huang J. C. (2000). Vascular endothelial growth factor and interleukin-6 in peritoneal fluid of women with endometriosis. *Fertility and Sterility*.

[46] Kim J. Y., Lee D. H., Joo J. K. (2009). Original article: Effects of peritoneal fluid from endometriosis patients on interferon-*γ*-induced protein-10 (CXCL10) and interleukin-8 (CXCL8) released by neutrophils and CD4+ T cells. *American Journal of Reproductive Immunology*.

[47] Szamatowicz J., Laudański P., Tomaszewska I., Szamatowicz M. (2002). Chemokine growth-regulated-alpha: a possible role in the pathogenesis of endometriosis. *Gynecological Endocrinology*.

[48] Na Y. J., Yang S. H., Baek D. W. (2006). Effects of peritoneal fluid from endometriosis patients on the release of vascular endothelial growth factor by neutrophils and monocytes. *Human Reproduction*.

[49] Bourlev V., Iljasova N., Adamyan L., Larsson A., Olovsson M. (2010). Signs of reduced angiogenic activity after surgical removal of deeply infiltrating endometriosis. *Fertility and Sterility*.

[50] Selam B., Kayisli U. A., Garcia-Velasco J. A., Akbas G. E., Arici A. (2002). Regulation of fas ligand expression by IL-8 in human endometrium. *The Journal of Clinical Endocrinology and Metabolism*.

[51] Suzumori N., Katano K., Suzumori K. (2004). Peritoneal fluid concentrations of epithelial neutrophil-activating peptide-78 correlate with the severity of endometriosis. *Fertility and Sterility*.

[52] Nasu K., Arima K., Kai K., Fujisawa K., Nishida M., Miyakawa I. (2001). Expression of epithelial neutrophil-activating peptide 78 in cultured human endometrial stromal cells. *Molecular Human Reproduction*.

[53] Mueller M. D., Mazzucchelli L., Buri C., Lebovic D. I., Dreher E., Taylor R. N. (2003). Epithelial neutrophil-activating peptide 78 concentrations are elevated in the peritoneal fluid of women with endometriosis. *Fertility and Sterility*.

[54] Bersinger N. A., Frischknecht F., Taylor R. N., Mueller M. D. (2008). Basal and cytokine-stimulated production of epithelial neutrophil activating peptide-78 (ENA-78) and interleukin-8 (IL-8) by cultured human endometrial epithelial and stromal cells. *Fertility and Sterility*.

[55] Bersinger N. A., Günthert A. R., McKinnon B., Johann S., Mueller M. D. (2011). Dose-response effect of interleukin (IL)-1*β*, tumour necrosis factor (TNF)-*α*, and interferon-*γ* on the in vitro production of epithelial neutrophil activating peptide-78 (ENA-78), IL-8, and IL-6 by human endometrial stromal cells. *Archives of Gynecology and Obstetrics*.

[56] Papayannopoulos V. (2018). Neutrophil extracellular traps in immunity and disease. *Nature Reviews. Immunology*.

[57] Berkes E., Oehmke F., Tinneberg H. R., Preissner K. T., Saffarzadeh M. (2014). Association of neutrophil extracellular traps with endometriosis-related chronic inflammation. *European Journal of Obstetrics, Gynecology, and Reproductive Biology*.

[58] Munrós J., Tàssies D., Reverter J. C. (2019). Circulating neutrophil extracellular traps are elevated in patients with deep infiltrating endometriosis. *Reproductive Sciences*.

[59] Fasciani A., D'Ambrogio G., Bocci G., Monti M., Genazzani A. R., Artini P. G. (2000). High concentrations of the vascular endothelial growth factor and interleukin-8 in ovarian endometriomata. *Molecular Human Reproduction*.

[60] Amatya N., Garg A. V., Gaffen S. L. (2017). IL-17 signaling: the yin and the yang. *Trends in Immunology*.

[61] Le Jan S., Plée J., Vallerand D. (2014). Innate immune cell-produced IL-17 sustains inflammation in bullous pemphigoid. *The Journal of Investigative Dermatology*.

[62] (1997). Revised American Society for Reproductive Medicine classification of endometriosis: 1996. *Fertility and Sterility*.

[63] Ahn S. H., Edwards A. K., Singh S. S., Young S. L., Lessey B. A., Tayade C. (2015). IL-17A contributes to the pathogenesis of endometriosis by triggering proinflammatory cytokines and angiogenic growth factors. *Journal of Immunology*.

[64] Takamura M., Osuga Y., Izumi G. (2012). Interleukin-17A is present in neutrophils in endometrioma and stimulates the secretion of growth-regulated oncogene-*α* (Gro-*α*) from endometrioma stromal cells. *Fertility and Sterility*.

[65] Ahn S. H., Khalaj K., Young S. L., Lessey B. A., Koti M., Tayade C. (2016). Immune-inflammation gene signatures in endometriosis patients. *Fertility and Sterility*.

[66] McDonald B., Pittman K., Menezes G. B. (2010). Intravascular danger signals guide neutrophils to sites of sterile inflammation. *Science*.

[67] Chen L., Wang X., Shu J., Xu S., Wu Q., Yu Y. (2019). Diagnostic value of serum D-dimer, CA125, and neutrophil-to-lymphocyte ratio in differentiating ovarian cancer and endometriosis. *International Journal of Gynaecology and Obstetrics: The Official Organ of the International Federation of Gynaecology and Obstetrics*.

[68] Lukács L., Kovács A. R., Pál L., Szűcs S., Kövér Á., Lampé R. (2021). Phagocyte function of peripheral neutrophil granulocytes and monocytes in endometriosis before and after surgery. *Journal Of Gynecology Obstetrics And Human Reproduction*.

[69] Libreros S., Iragavarapu-Charyulu V. (2015). YKL-40/CHI3L1 drives inflammation on the road of tumor progression. *Journal of Leukocyte Biology*.

[70] Tuten A., Kucur M., Imamoglu M. (2014). Serum YKL-40 levels are altered in endometriosis. *Gynecological Endocrinology*.

[71] Ning X., Wang W. M., Jin H. Z. (2022). Low-density granulocytes in immune-mediated inflammatory diseases. *Journal of Immunology Research*.

[72] Denny M. F., Yalavarthi S., Zhao W. (2010). A distinct subset of proinflammatory neutrophils isolated from patients with systemic lupus erythematosus induces vascular damage and synthesizes type I IFNs. *Journal of Immunology*.

[73] Rahman S., Sagar D., Hanna R. N. (2019). Low-density granulocytes activate T cells and demonstrate a non-suppressive role in systemic lupus erythematosus. *Annals of the Rheumatic Diseases*.

[74] Hassani M., Hellebrekers P., Chen N. (2020). On the origin of low-density neutrophils. *Journal of Leukocyte Biology*.

[75] Lin Y. J., Lai M. D., Lei H. Y., Wing L. Y. (2006). Neutrophils and macrophages promote angiogenesis in the early stage of endometriosis in a mouse model. *Endocrinology*.

[76] Takamura M., Koga K., Izumi G. (2016). Neutrophil depletion reduces endometriotic lesion formation in mice. *American Journal of Reproductive Immunology*.

[77] Deniset J. F., Surewaard B. G., Lee W. Y., Kubes P. (2017). Splenic Ly6G (high) mature and Ly6G (int) immature neutrophils contribute to eradication of S. pneumoniae. *The Journal of Experimental Medicine*.

[78] Guo F., He Y., Fan Y. (2021). G-CSF and IL-6 may be involved in formation of endometriosis lesions by increasing the expression of angiogenic factors in neutrophils. *Molecular Human Reproduction*.

[79] Burns K. A., Thomas S. Y., Hamilton K. J., Young S. L., Cook D. N., Korach K. S. (2018). Early endometriosis in females is directed by immune-mediated estrogen receptor *α* and IL-6 cross-talk. *Endocrinology*.

[80] Lim C. L., Or Y. Z., Ong Z. (2020). Estrogen exacerbates mammary involution through neutrophil-dependent and -independent mechanism. *eLife*.

[81] Tokmak A., Yildirim G., Öztaş E. (2016). Use of neutrophil-to-lymphocyte ratio combined with CA-125 to distinguish endometriomas from other benign ovarian cysts. *Reproductive Sciences*.

[82] Yang H., Lang J. H., Zhu L., Wang S., Sha G. H., Zhang Y. (2013). Diagnostic value of the neutrophil-to-lymphocyte ratio and the combination of serum CA-125 for stages III and IV endometriosis. *Chinese Medical Journal*.

[83] Moini A., Ghanaat M., Hosseini R., Rastad H., Hosseini L. (2021). Evaluating hematological parameters in women with endometriosis. *Journal of obstetrics and gynaecology: the journal of the Institute of Obstetrics and Gynaecology*.

[84] Viganò P., Ottolina J., Sarais V., Rebonato G., Somigliana E., Candiani M. (2018). Coagulation status in women with endometriosis. *Reproductive Sciences*.

[85] Monsanto S. P., Edwards A. K., Zhou J. (2016). Surgical removal of endometriotic lesions alters local and systemic proinflammatory cytokines in endometriosis patients. *Fertility and Sterility*.

[86] Kalu E., Sumar N., Giannopoulos T. (2007). Cytokine profiles in serum and peritoneal fluid from infertile women with and without endometriosis. *The Journal of Obstetrics and Gynaecology Research*.

[87] Tarokh M., Ghaffari Novin M., Poordast T. (2019). Serum and peritoneal fluid cytokine profiles in infertile women with endometriosis. *Iranian Journal of Immunology*.

